# An Exploratory Study of Sleep Quality After Lung Transplantation Using the Pittsburgh Sleep Quality Index

**DOI:** 10.1177/15269248251349752

**Published:** 2025-06-17

**Authors:** Jane Simanovski, Jody Ralph, Sherry Morrell

**Affiliations:** 1 Faculty of Nursing, 8637 University of Windsor, Windsor, Ontario, Canada; 2 Transplant Institute, Henry Ford Hospital, Detroit, MI, USA

**Keywords:** statistics, descriptive, lung transplantation, sleep quality, subjective sleep quality, Pittsburgh Sleep Quality Index, PSQI, sleep

## Abstract

**Introduction:** Sleep is essential for maintaining optimal physical and mental health as it supports crucial functions such as cognition, immune system regulation, and overall well-being. A growing emphasis on the importance of sleep warrants an investigation of sleep quality after lung transplantation. **Research Question:** What is the overall prevalence, nature, and severity of patient-reported disrupted sleep quality after lung transplantation using the Pittsburgh Sleep Quality Index (PSQI)? **Design:** This study employed a single-site, exploratory, cross-sectional descriptive design involving lung transplant recipients who completed an anonymous survey. Sleep quality was assessed using the PSQI scale. Additionally, participants provided self-reported data on demographic and transplant-related variables. **Results:** The response rate was 38.4% (61/158) and 64% of the respondents (39/61) demonstrated PSQI >5 with a mean PSQI score of 8.07 (SD = 4.5), suggestive of poor sleep quality. Lung transplant recipients reported difficulties across all components of sleep quality with more challenges in the categories of sleep duration, sleep latency, sleep efficiency, and the use of sleep medications. **Conclusion:** The prevalence of poor subjective sleep quality among lung transplant recipients highlighted the importance of continued investigation into this phenomenon. Further research employing standardized measures, larger sample sizes, and longitudinal study designs is warranted to enhance understanding of poor sleep post-lung transplant. Such endeavors are crucial for informing the development of effective assessment strategies and interventions aimed at improving sleep outcomes in patients after lung transplantation.

## Background

Lung transplantation stands as the final resort for individuals grappling with idiopathic pulmonary fibrosis, chronic obstructive pulmonary disease, cystic fibrosis, and other end-stage lung diseases.^1^ According to the International Society for Heart and Lung Transplantation registry, there were 67 861 primary adult lung transplants performed worldwide between 1992 and 2024.^1^ Despite advancements in surgical and immunosuppressive methods, median graft survival, defined as freedom from retransplantation or death after lung transplant, remains low averaging 5.8 years.^2^ In addition, by 10 years after the initial lung transplant, 4.3% of adult lung transplant recipients had received a second transplant.^2^ Given the scarcity of available organs, there is an intensified emphasis on enhancing posttransplant survival rates and quality of life by addressing factors contributing to overall health outcomes.^3^ Sleep was recognized as a vital mechanism for maintaining overall health by facilitating the repair of multiple physiological processes.^4^ Surprisingly, little is understood about sleep and sleep quality post-lung transplantation. The seminal study by Buysse et al^5^ pioneered the assessment of subjective sleep quality with the Pittsburgh Sleep Quality Index (PSQI), suggesting that poor sleep quality and disruptions can significantly impact quality of life and may correlate with emotional and physical ailments. A recent publication by Simanovski et al^6^ highlighted several factors linked to poor sleep in lung transplant recipients but information on specific sleep indicators was lacking. The Sleep Quality Panel^7^ reached a consensus that indicators such as the time it takes to fall asleep (known as sleep latency), the frequency of awakenings lasting longer than 5 min, the duration of wakefulness after initially falling asleep, and overall sleep efficiency (ratio of the time asleep vs the time spent in bed) were suitable measures of sleep quality for people of all ages.^7^

Extended hospital stays, readmissions, adverse effects from the immunosuppressive medications in addition to complications such as primary graft dysfunction, acute cellular and humoral rejections, infections, diabetes, cardiovascular disease, and gastrointestinal comorbidities characterize the lung transplant journey.^1,6,8^ A scoping review on sleep quality after lung transplantation identified only 12 sources (including abstracts) that addressed sleep quality or its components in lung transplant recipients, with 4 of using PSQI as a measure of sleep quality. This review indicated that 32% to 81% of lung transplant patients experienced poor sleep quality based on the PSQI scoring.^8^ A systematic review on sleep quality after all solid organ transplants by Cordoza et al^9^ appraised 44 studies with only 4 focusing on lung transplant recipients. Consistent with the scoping review,^8^ Cordoza et al^9^ found that 46.3% of lung transplant recipients suffer from poor sleep quality.

Considering the importance of sleep on health and the identified gap in the lung transplant literature, the purpose of this study was to characterize the nature of subjective sleep quality after lung transplantation. The study aimed to describe the prevalence, nature, and severity of patient-reported disrupted sleep quality after lung transplantation using the overall and component scores of the PSQI.

## Methods

### Design

This was a descriptive analysis of the PSQI scoring from lung transplant recipients who participated in the larger study exploring associations with poor sleep.^6^ This quantitative investigation constituted a single-site, cross-sectional, observational, descriptive study of lung transplant recipients under the care of a transplant center in the United States's Midwest region. The study obtained ethics approval from the institutional review board. As the survey administered to the subjects maintained anonymity, the research was deemed exempt status and qualified for a waiver for documenting informed consent under the institutional review board. Although each participant received necessary consent information along with the survey, obtaining the participant's signature on the informed consent document was not required for the study team; completion of the survey indicated participant consent.

### Setting and Population

Recipients of lung transplants at a well-established, medium-size transplant center in the Midwest that performed over 450 lung transplants since launching its program in 1994^10^ were invited to participate in this anonymous survey. The center's demographic and survival data are consistent with the national trends as reported to the United States Organ Procurement and Transplantation Network.^10^

### Sampling

The study used the following inclusion criteria for eligibility to participate: (a) individuals who had received a lung transplant and were being monitored at the lung transplant center, (b) older than 18 who were able to understand, read, and write in English, and (c) capable of giving informed consent. Exclusion criteria included those who did not meet inclusion criteria and those critically ill requiring admission in the intensive care unit due to treatments with life-sustaining invasive therapies such as mechanical ventilation, vasoactive medications, and/or extracorporeal life support. Subjects were enrolled between May 2022 and September 2022. As part of the recruitment strategy, upon completing the survey, participants were informed that they were eligible to receive a $15 Target Gift Card, a token of appreciation for completing the survey.

### Data Collection

#### Sample Characteristics

The participant characteristics were collected through self-reported data, which included demographic details (such as age, sex at birth, marital status, and race) as well as information on transplant laterality, presence of comorbidities (ie, diabetes, cardiovascular disease, acid reflux, and diagnosed sleep disorders), history of hospitalization, treatment of rejection, and the use substances such as alcohol, cannabinoids, and herbs to assist with sleep in the past 30 days.

#### Subjective Sleep Quality: PSQI

Subjective sleep quality was assessed using the global score from the PSQI.^5^ The questionnaire consists of 19 items and evaluates the global sleep quality score and 7 component scores: sleep quality, sleep latency, sleep duration, sleep efficiency, sleep disturbances (eg, restroom usage), use of sleeping medications, and daytime dysfunction.^5^ These component scores are weighed on a 0 to 3 scale and then summed to determine a global PSQI score.^5^ A global PSQI score of 5 or higher is indicative of poor sleep quality.^5^ While not validated explicitly in the lung transplant population, the psychometric properties of the PSQI have been examined in similar patient groups, such as bone marrow and renal transplant recipients.^11^ These studies support the internal consistency, reliability, and construct validity of the PSQI. As measured by Cronbach's α coefficients, internal consistency has been reported to range from 0.80 to 0.83 across various populations, including solid organ transplant recipients.^11^ Permission to use the PSQI in this study was granted by its developers.

### Statistical Analysis

SPSS Version 28.0 (IBM) was used for analysis. Before the analysis, data were screened for the presence and pattern of missing data. There was a small amount of missing data within the dataset. REDCap registered 12 users who did not input the study data as they did not move beyond the first question. Given that they did not complete any of the study instruments, they were deleted from the sample. There were 3 data points that were skipped among all the PSQI items. The valid sample mean was used to populate these cells. Otherwise, this sample involved a complete case analysis. Measures of frequency and central tendency are reported including descriptive statistics, means, standard deviation, and minimum/maximum values for continuous variables (interval/ratio level) and frequencies and percentages for categorical variables (nominal/ratio level).

#### Procedure

Data collection took place between May 2022 and September 2022. Potential subjects were approached by the principal investigator, either during their scheduled appointments at the transplant clinic or through a flyer sent to their home address. Every effort was made to engage subjects face-to-face using a standardized recruitment script. Additionally, targeted communication to lung transplant recipients was disseminated electronically via the hospital patient communication portal in EPIC (Epic Systems Corporation), including an information letter, a link to the survey, and a recruitment flyer featuring a QR code, a 2-dimensional code readable by a camera phone allowing quick and convenient access to the electronic survey.

Study variables were collected and managed using the REDCap electronic data capture tool (Vanderbilt University). Surveys were anonymous and typically required approximately 20 min to complete the questionnaire it its entirety. A paper version of the survey was also made available upon request, along with a prepaid addressed envelope for the mail return to the principal investigator. Upon receiving the paper survey via mail, the principal investigator manually input the data into the REDCap project. A coinvestigator verified the accuracy of the data entry. No identifying information was collected on either the electronic or paper versions.

## Results

### Sample Characteristics

Of the 159 subjects who met the inclusion criteria to participate in the study, 61 completed a questionnaire, yielding a response rate of 38.4% (N = 61). As shown in **Table 1**, the sample was largely male (N = 41, 67.2%), married (N = 42, 68.9%), White (N = 47, 77.0%), and 30 to 77 years of age (M = 61.46). Most of the participants were within 3 years of transplant (N = 40; 66.7%) and have received a bilateral lung transplant (N = 57; 93.4%). Many subjected self-reported comorbidities such as diabetes (N = 23, 37.7%), cardiovascular disease (N = 22, 36.1%), acid reflux (N = 30, 49.2%), and diagnosed sleep disorders like sleep apnea (N = 13, 21.37%). Many participants identified the following stressors in the preceding 30 days: hospitalization (N = 14, 23%), treatment of rejection (N = 4, 6.6%), and illness of a family member (N = 10, 16.4%). While no subjects reported using alcohol (N = 0, 0%), a few participants reported the use of cannabis (N = 3; 4.9%) and herbal preparations (N = 3; 4.9%).

**Table 1.** Sample Characteristics.

Measure	N (%)	Mean (SD)
Respondent age		61.46 (10.16)
Birth sex		
Male	41 (67.2)	
Relationship status		
Married	42 (68.9)	
Single	11 (18)	
Divorced	4 (6.6)	
Living with a partner	4 (6.6)	
Self-identified race		
White	47 (77.0)	
Black	7 (11.5)	
Hispanic ethnicity	2 (3.3)	
Other	5 (8.2)	
Years elapsed since transplant		
0-3 years	40 (66.7)	
Laterality of transplant		
Bilateral	57 (93.4)	
Self-reported comorbidities		
Diabetes mellitus	23 (37.7)	
Cardiovascular disease	22 (36.1)	
Gastroesophageal reflux disease	30 (49.2)	
Diagnosed sleep disorder (eg, sleep apnea)	13 (21.3)	
Self-reported stressors (responded yes)		
Hospitalized in the last 30 days	14 (23.0)	
Treated for acute rejection in the last 30 days	4 (6.6)	
Ill family member	10 (16.4)	
Self-reported use of substances to assist with sleep in the past 30 days (responded no)		
Use of alcohol (no)	0 (0)	
Use of cannabinoids (no)	58 (95.1)	
Use of herbs (no)	58 (95.1)	

### Pittsburgh Sleep Quality Index and Subscales

**Figure 1.**
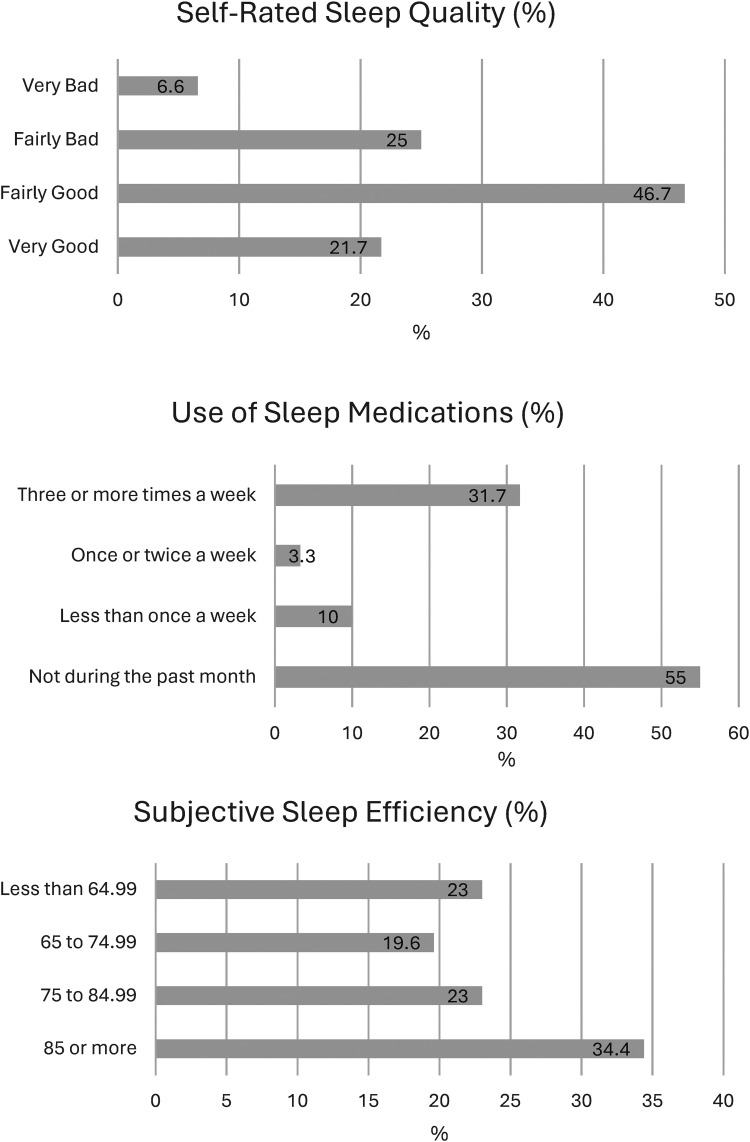

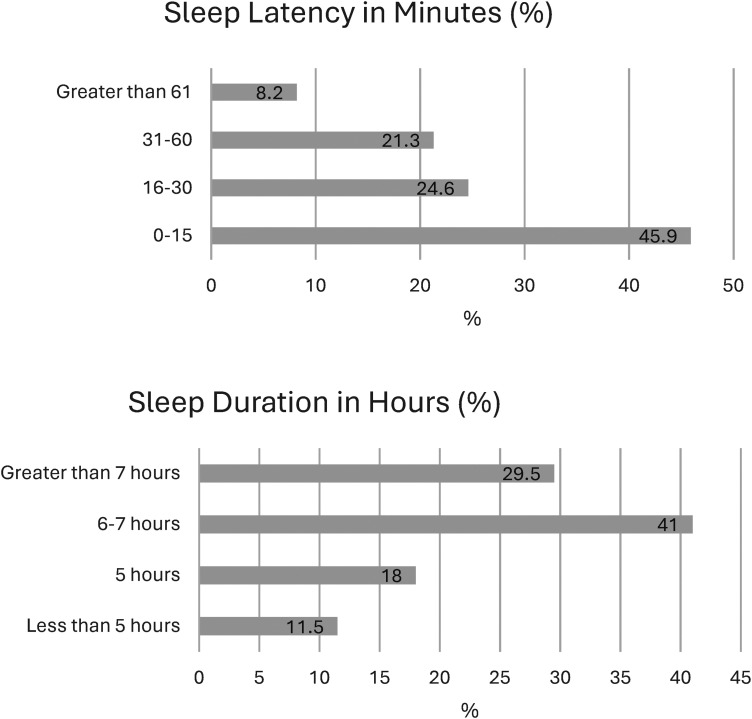
Descriptive analysis of sleep quality components based on PSQI scale (N = 61). Abbreviation: PSQI, Pittsburgh Sleep Quality Index.

There were 64% (N = 39) of participants with PSQI scores greater than 5 indicative of poor sleep as operationalized in the seminal work by Buysse et al.^5^ Data within **Table 2** show that the mean PSQI score in this sample of participants was 8.07 (SD = 4.5). **Table 2** and **Figure 1** represent a descriptive analysis of overall sleep quality and its components. These scores collectively suggest some degree of sleep difficulty across all measured components. A substantial proportion of participants exceeded benchmarks for poor sleep. For instance, approximately one-third of the sample reported increased sleep latency or more than 30 min to fall asleep at bedtime (N = 18, 29.5%) and sleeping fewer than 6 h at night (N = 18, 29.5%). A further two-thirds lacked sleep efficiency whereby these individuals reported less than 85% of the total time in bed was spent sleeping (N = 40, 65.6%). Approximately one-third of the lung transplant recipients rated their sleep quality as fairly bad or very bad (N = 19, 31.7%). Almost half of the study sample reported using sleep medications at least once in the last month (N = 27, 45%).

**Table 2.** Overall Sleep Quality and its Components Based on PSQI (N = 61).^a^

Variable	Mean (SD)	Minimum/Maximum
Global PSQI score	8.07 (4.45)	1.00-19.00
Sleep disturbance	1.52 (0.566)	1.00-3.00
Duration of sleep	0.92 (1.07)	0.00-3.00
Sleep latency	1.38 (1.08)	0.00-3.00
Day dysfunction	0.67 (0.65)	0.00-3.00
Sleep efficiency	1.31 (1.78)	0.00-3.00
Overall sleep quality	1.16 (0.84)	0.00-3.00
Use of sleep medications	1.1 (1.36)	0.00-3.00

Abbreviations: PSQI, Pittsburgh Sleep Quality Index; SD, standard deviation.

^a^ A global PSQI score of 5 or higher is indicative of poor sleep quality.

**Figure 2.**
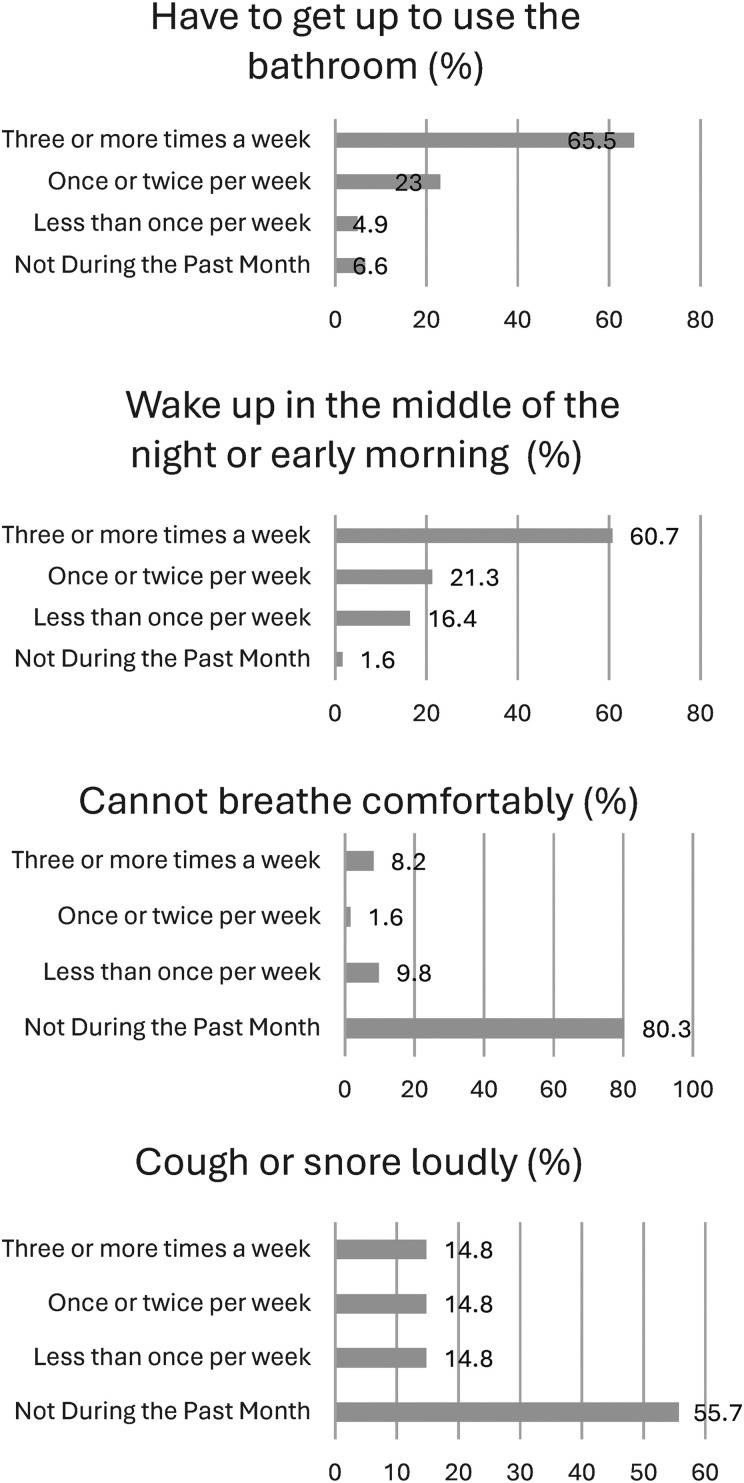

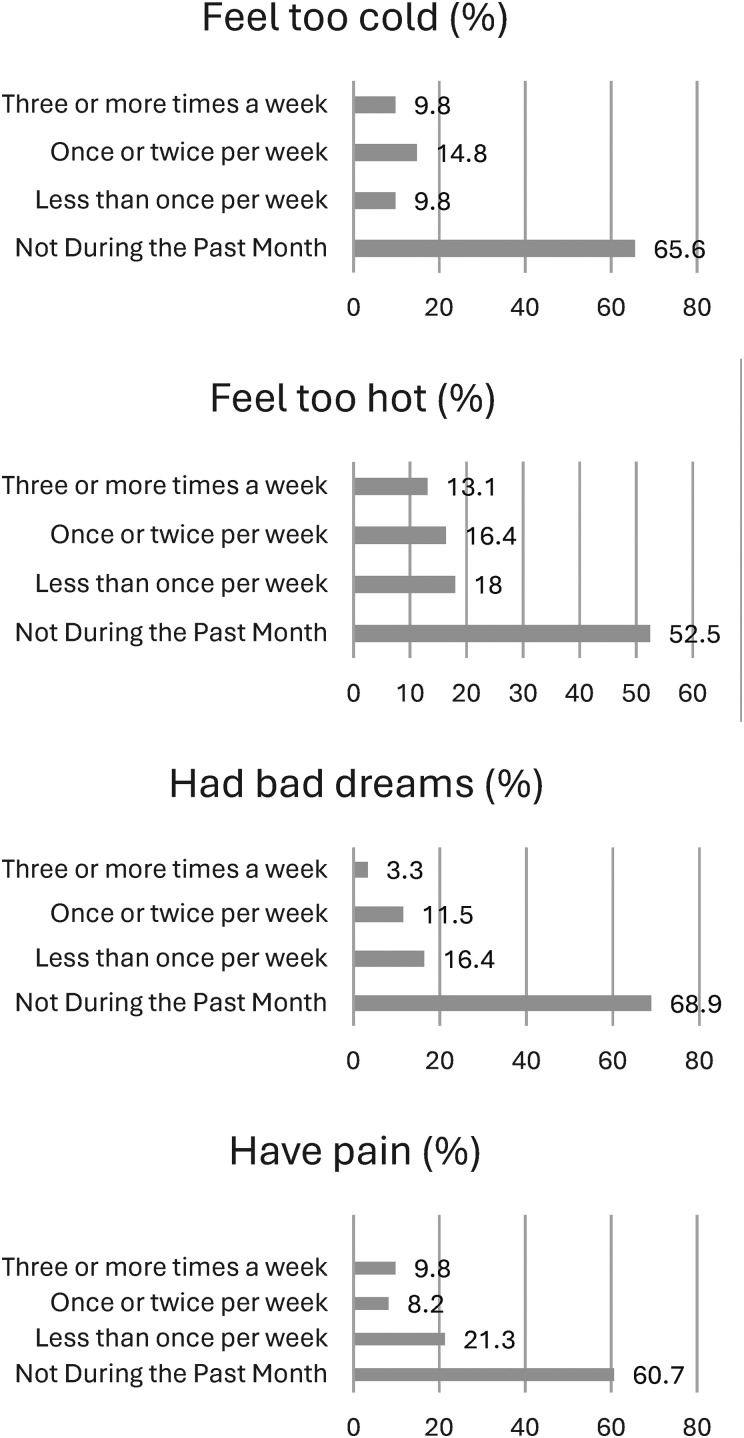
Subjective sleep disturbances based on PSQI (N = 61). Abbreviation: PSQI, Pittsburgh Sleep Quality Index.

The PSQI includes reasons for sleep disturbances, presented in **Figure 2**. The following reasons reflect the proportion of the current sample who reported these problems more than once per week: Waking up in the middle of the night or early morning (N = 50, 82%), having to get up to use the bathroom (N = 54, 88.6%), not breathe comfortably (N = 6, 9.8%), cough or snore loudly (N = 18, 29.6%), feel too cold (N = 15, 24.6%), feel too hot (N = 18, 29.5%), and had bad dreams (N = 9, 14.8%). Other reasons, which included handwritten responses, are summarized in **Table 3**.

**Table 3.** Descriptive Analysis of Other Reasons for Sleep Disturbances (N = 14).

Response	Frequency of occurrence reported
Wake up and roll over every couple of hours	Three or more times a week
Too much on my mind	Three or more times a week
Restlessnes	Three or more times a week
Pain from incision. I have to sleep on my back when normally I'm a side sleeper	Three or more times a week
Migraine/pressure in head	Three or more times a week
I toss and turn and wake up a ton most nights. I feel restless most times	Three or more times a week
Wide Awake	Once or twice per week
Wake to go to the bathroom because I have a colostomy bag. The reattachment surgery to be completed within 3 months	Once or twice per week
I sometimes have night sweats that wake me up because of the bed being soaked	Once or twice per week
Took naps during the day	Less than once per week
Sleeping in a weird place such as a hotel	Less than once per week
Restless legs cramping in legs	Less than once per week
Anxiety plays a large role. Being dependent on an Oxygen concentrator for 5 years and then not having it was a difficult adjustment. Initially this caused me to wake up panicked frequently	Less than once per week
Back pain due to Vietnam	Not during the past month

## Discussion

There is limited literature investigating sleep quality after lung transplantation. This exploratory, cross-sectional study aimed to describe subjective sleep quality in 61 lung transplant recipients using the PSQI. While the inferential analyses were reported previously by Simanovski et al,^6^ the descriptive statistics indicate that more than two-thirds of the study participants (N = 39, 64%) exhibited poor sleep quality within the current sample, as determined by a PSQI cutoff score exceeding 5. This aligns with previous research using the PSQI to assess subjective sleep quality, where other authors have reported the presence of poor sleep in 32% to 81% of lung transplant recipients.^12-15^ Two studies^14,15^ used a PSQI cutoff score greater than 5, while Fatigati et al^12^ and Reilly-Spong et al^13^ employed a cutoff score greater than 8.

The PSQI component scores demonstrated that poor self-reported sleep may be due to inadequate sleep duration and increased sleep latency experienced in one-third of the sample; this was consistent with studies by Fatigati et al^12^ and Sawhney et al.^14^ Sleep duration may vary across the lifespan.^16,17^ An overview of systematic reviews on sleep duration in adults aged 18 years and older summarized the evidence from 36 systematic reviews indicating that sleep duration of 7 to 8 h per day was most positively associated with health.^16^ One-third of this study's sample reported sleeping fewer than 6 h at night (N = 18, 29.5%). The National Sleep Foundation recommends 7 to 9 h of daily sleep for adults aged 18 to 64 and 7 to 8 h for adults older than 65.^17^ A review by Chaput et al^16^ found that there is a clear association between short sleep and adverse health outcomes. Most of the evidence in their review came from the self-reported sleep questionnaires and it is unclear whether this correlates well with objective sleep measures.^16^ Similarly, studies on sleep quality utilizing polysomnography or actigraphy in lung transplant populations are scarce, and more research is needed employing both objective and subjective measures.^8^

Sleep latency refers to the time it takes for individuals to fall asleep.^7^ Similar to sleep duration, it also fluctuates across the lifespan^7,18^; it may be influenced by substance use and chronic health conditions.^19^ Generally, sleep latency under 30 min is a good indicator of healthy sleep quality^7,19^; almost one-third of the subjects in this study self-reported sleep latency greater than 30 min. Although none of the subjects reported the use of alcohol, many of them reported significant comorbidities acquired before or after their transplant, which are known to be associated with poor sleep.

Sleep latency was also linked to sleep efficiency as both measures provide information about how well individuals slept. Prolonged sleep latency can decrease sleep efficiency.^19^ Sleep efficiency calculates the total time in bed that is spent in sleep.^7,19^ For example, the sleep efficiency of an individual who spends 8 h in bed but asleep for only 4 h is equivalent to 50%. Ideally, the healthy sleep efficiency should be 85% or more for optimal health benefits.^17^ In this study, two-thirds of the current sample lacked sleep efficiency, similar to findings by Reilly-Spong et al^13^ and Sawhney et al.^14^ Many factors, including aging, can impact sleep efficiency including sociodemographic variables and physical and mental health concerns.^20^ The mean age of this study's subjects was 61.46 years old, with many individuals self-reporting the presence of diabetes, cardiovascular disease, gastroesophageal reflux disease, and diagnosed sleep disorders, which all potentially may have an impact on the efficiency of sleep. Individuals can experience low sleep efficiency due to sleep disturbances, which were also abundant in this study's sample.

As depicted in Figure 2, the most common sleep disturbances reported in three-quarters of the sample included waking up in the middle of the night or early morning and getting up to use the bathroom. Pathology, medications, and symptoms associated with physiological and psychological conditions often disrupt normal sleep physiology.^7,9^ Any of these stressors can disturb sleep quality by prolonging the time it takes to fall asleep (sleep latency), causing multiple awakenings at night, which can also shorten sleep duration and impair sleep.^9^ Medical regimens post-lung transplantation are complex, requiring immaculate timing for immunosuppression administration and complex regiment (eg, tracheostomy care, administration of inhaled medications, and management of enteral feeding). Many patients require the use of diuretics, which may lead to nocturia, and steroids that interfere with the sleep cycle. Almost half of the study sample reported using sleep medications at least once in the last month, consistent with findings by Tokuno et al.^15^ Given the increased prevalence of poor sleep in our society, many people resort to pharmacological sleep aids in an attempt to initiate or maintain sleep.^21^ A recent study of 484 961 adults noted that the use of sleeping pills was associated with an increased risk of mortality and shortened life expectancy,^21^ which is essential to keep in mind given an already limited survival after lung transplantation.

### Limitations

This study has limitations worth noting. Firstly, this was a single-center observational study using a small convenience sample, potentially introducing selection bias and limiting the generalizability of its findings. Future research should involve multiple transplant centers and larger groups for broader insights. Secondly, the sleep quality assessment was done at one point, missing potential variations over time. Longitudinal studies are needed to account for the dynamic nature of sleep. Thirdly, reliance on self-report questionnaires may introduce response and recall biases; integrating objective measures in future research could improve the reliability of findings. Similarly, this study may also be subject to nonresponse bias, as the characteristics of individuals who completed the survey could differ systematically from those who did not respond. Such differences may influence the validity and generalizability of the findings, particularly if nonresponders experience different sleep parameters and experiences relevant to the study outcomes. Finally, this study's descriptive nature did not allow for examining the potential causal relationships between demographic variables and PSQI scores that were reported in an earlier publication.^6^ Despite these limitations, the study offers unique insight into sleep quality post-lung transplantation, a topic warranting more attention in an era where sleep is recognized as a biological necessity vital for health, well-being, and safety.^4^

## Conclusion

The results of this study offer insights into the increased prevalence and nature of poor sleep after lung transplantation. It highlights the importance of continued investigation into this phenomenon. Further research employing standardized measures, larger sample sizes, and longitudinal study designs is warranted to enhance understanding of poor sleep post-lung transplant. Such endeavors are crucial for informing the development of effective assessment strategies and interventions to improve sleep outcomes in patients after lung transplantation. Clinicians should also routinely inquire about sleep quality with each patient encounter. Further development of patient-reported outcome measures focusing on clinical assessment of sleep is warranted given the complexity of the concept and lack of standardized clinical tools.
